# Current and Future Applications of Biomedical Engineering for Proteomic Profiling: Predictive Biomarkers in Neuro-Traumatology

**DOI:** 10.3390/medicines5010019

**Published:** 2018-02-05

**Authors:** Mario Ganau, Nikolaos Syrmos, Marco Paris, Laura Ganau, Gianfranco K.I. Ligarotti, Ali Moghaddamjou, Salvatore Chibbaro, Andrea Soddu, Rossano Ambu, Lara Prisco

**Affiliations:** 1 Department of Neurosurgery, Toronto Western Hospital, University of Toronto, Toronto, ON M5T 2S8, Canada; alim937@hotmail.com; 2 School of Medicine, University of Cagliari, 09124 Cagliari, Italy; lolly26it@yahoo.it (L.G.); amburo@unica.it (R.A.); 3 School of Medicine, Aristotle University of Thessaloniki, 54623 Thessaloniki, Greece; milanako76@yahoo.gr; 4 National Hospital for Neurology and Neurosurgery, University College London, London WC1N 3BG, UK; marco.paris@uclh.nhs.uk; 5 Fondazione IRCCS IstitutoNeurologico “Carlo Besta”, 20133 Milano, Italy; gianfrancokiligarotti@gmail.com; 6 Division of Neurosurgery, University of Strasbourg, 67000 Strasbourg, France; schibbaro@hotmail.com; 7 Brain and Mind Institute, Physics & Astronomy Department, Western University, London, ON N6A 3K7, Canada; asoddu@uwo.ca; 8 John Radcliffe Hospital, Oxford University, Oxford OX3 9DU, UK; lara.prisco@ndcn.ox.ac.uk

**Keywords:** traumatic brain injury, biomarkers, proteomics, microRNA, mass spectroscopy

## Abstract

This systematic review aims to summarize the impact of nanotechnology and biomedical engineering in defining clinically meaningful predictive biomarkers in patients with traumatic brain injury (TBI), a critical worldwide health problem with an estimated 10 billion people affected annually worldwide. Data were collected through a review of the existing English literature performed on Scopus, MEDLINE, MEDLINE in Process, EMBASE, and/or Cochrane Central Register of Controlled Trials. Only experimental articles revolving around the management of TBI, in which the role of new devices based on innovative discoveries coming from the field of nanotechnology and biomedical engineering were highlighted, have been included and analyzed in this study. Based on theresults gathered from this research on innovative methods for genomics, epigenomics, and proteomics, their future application in this field seems promising. Despite the outstanding technical challenges of identifying reliable biosignatures for TBI and the mixed nature of studies herein described (single cells proteomics, biofilms, sensors, etc.), the clinical implementation of those discoveries will allow us to gain confidence in the use of advanced neuromonitoring modalities with a potential dramatic improvement in the management of those patients.

## 1. Introduction

Traumatic brain injury (TBI) represents a critical worldwide health problem and, despite remarkable advances in medical and surgical management, its prognosis remains a major challenge of modern healthcare systems [1,2].

With an estimated 10 million people affected annually by TBI worldwide, it is predicted by the World Health Organization (WHO) that by the year 2020, TBI will surpass many diseases to become the third leading cause of global mortality and disability [3]. The CDC has recently reported that nearly one third (30.5%) of deaths associated with traumas include a diagnosis of TBI, and there are an estimated 5.3 million U.S. residents living with TBI-related disabilities; already in 2010, the economic costs resulting from TBI were estimated at $76.5 billion/year, including $11.5 billion for direct medical costs and $64.8 billion for indirect costs (i.e., lost wages, lost productivity, and nonmedical expenditures) [4].

The clinical spectrum of TBI can range from mild injuries, such as asymptomatic sub-concussive blows or symptomatic concussion, to more severe conditions leading to a comatose state. The most common cause for severe TBI is certainly represented by road traffic accidents, falls, and penetrating gunshot injuries; nonetheless also practicing popular sports, such as American football, ice hockey, boxing, martial arts, rugby, and even soccer, horse riding, or parachuting, carries a significant risk of exposure to mild to severe brain trauma [1,5,6].

As a result of an initial injury, a number of complex pathological mechanisms that include elements of excitotoxicity, oxidative damage, and cerebrovascular derangements may be triggered. Cellular, sub-cellular, and molecular pathological processes that become activated include decreased mitochondrial respiratory capacity (thus, energy failure), damage of liposomes, activated mechanisms of apoptotic and non-apoptotic delayed cell death, triggered cascades of inflammation, and protein degradation [7,8]. Additionally, several metabolic changes, known to occur after TBI, alter the metabolization of amino acids, carbohydrates, and lipids, with consequences affecting multiple organs and not only the brain [9]. Since these systemic metabolic alterations accompanying inflammation contribute to and/or aggravate the impaired energy production and neurotransmitter synthesis in the brain, and significantly worsen the injury outcome, in recent years neuro-traumatologists attempted to (a) elucidate the complex mechanisms of TBI progression; and (b) seek acute and chronic biomarkers helpful in optimizing TBI prognosis and management.

Through a systematic review of the current English literature, we will offer an overview of how improvements in the state of the art of many methodologies for genomics, epigenomics, and proteomics testing are rapidly finding a place in modern medicine, by improving the understanding and management of patients with TBI. This work will therefore focus on the latest discoveries in quantitative neuroscience, specifically those that hold the promise to foster the field of preventive and personal medicine in neuro-traumatology.

## 2. Materials and Methods

This article aims at providing the readers with an overview of all the most recent studies in which the role of new devices based on innovative discoveries coming from the field of nanotechnology and biomedical engineering were highlighted with regards to proteomic profiling in patients with TBI.

*Study Characteristics*: This article focuses on basic sciences and clinical studies that used innovative genomics, epigenomics, and proteomic techniques to identify clinically meaningful biomarkers or biosignatures with the potential to predict clinical and surgical outcomes in patients with TBI. While we have included any type of experimental paper (including animal and biomechanical studies), the following types of articles were excluded from this review: reviews, letters, editorials/commentaries, meeting abstracts, conference reports, and books.

*Information Sources*: A systematic search of MEDLINE, MEDLINE in Process, EMBASE, and/or Cochrane Central Register of Controlled Trials was conducted to identify relevant studies.

*Search Strategy*: We developed a search strategy with a librarian who specializes in neuroscience research. The strategy was first developed in MEDLINE and then appropriately modified for the other databases. The following search terms were used at the time all databases were interrogated (November 2017): “Traumatic Brain Injury” and “Nanotechnology” or “Biomedical Engineering”, and “biomarkers” or “biosignatures”, and “clinical outcomes” or “surgical outcomes”. Only studies written in English were considered for inclusion, with no other limits applied in terms of type of study (basic science/clinical study). The results of this search were thoroughly reviewed: initially by four authors with extensive experience in basic laboratory studies, and finally validated by four authors with clinical expertise in the management of TBI. A final check by all authors was carried out to ensure that only experimental studies providing (a) a materials and methods section with a detailed description of new screening methods based on nanotechnology or biomedical engineering; and (b) a result section describing their correlation with clinical and surgical outcomes, had been retained for further analysis and reporting in this systematic review.

*Data Extraction and Synthesis*: The following data were extracted from each included article: study design, publication date, samples used for identification of biomarkers/biosignatures, and clinical/surgical outcomes included in the study.

*Reporting*: The results of this review were formatted based on the Preferred Reporting Items for Systematic Reviews and Meta-Analyses (PRISMA) statement [10]. 

## 3. Results

**Figure 1:**
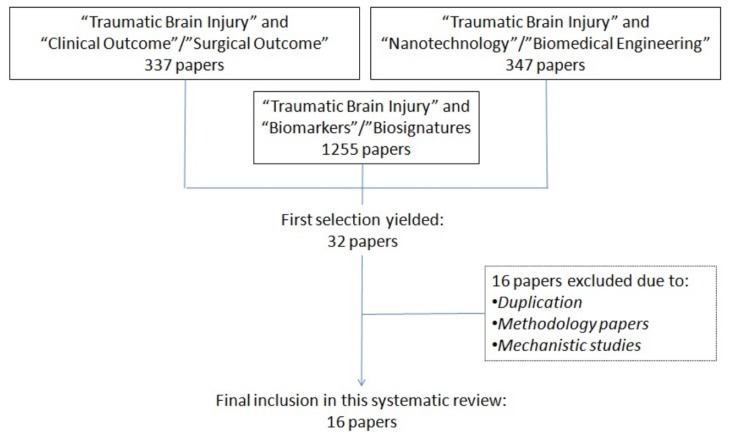
Selection process of suitable articles to be analyzed in this systematic review.

The initial search of the literature yielded to 1939 articles, which were then screened in two consecutive rounds by two groups of four experts (scientists for the first round and clinicians for the second round) involved in this study. This triage of the literature led to an initial selection of 32 papers, out of which 16 were excluded due to duplication of the papers identified, or because the articles dealt with the description of a methodology or the description of physiological/pathological pathways but eventually failed to provide a correlation between the identification of a biomarker/biosignature and the related clinical or surgical outcome. A diagram summarizing the design of this systematic review is provided (Figure 1).

The 16 articles selected fall into two main categories: 8 clinical papers and 8 laboratory studies on animal models of TBI [11,12,13,14,15,16,17,18,19,20,21,22,23,24,25,26]. Briefly, the 8 clinical studies identified have described four main technologies for identification of predictive biomarkers: (a) Microvescicle/Exosome, (b) MicroRNA, (c) Mass Spectroscopy, and (d) Multiplexing and Immunoassays [11,12,13,14,15,16,17,18]. On the other hand, the methodology of the 8 laboratory studies was based on (a) MicroRNA, (b) Mass Spectroscopy, and (c) Multiplexing and Immunoassays; those latter investigated several different animal models of TBI ranging from mild to severe TBI including blunt traumas and diffuse axonal injuries; a wide range of animals were investigated in those studies, they included small size animals such as mice and rats but also rabbits and larger size animals such as pigs [19,20,21,22,23,24,25,26].

A summary for each of the 16 articles identified in this systematic review, including research setting, details of the cohort/sample studied, analytical methodology, biomarker(s) identified, most significant results, and related externalities in term of prognosis, is provided in Table 1 and Table 2, respectively.

## 4. Discussion

Diagnostics in neuro-trauma investigate the numerous changes suddenly occurring in the central nervous system (CNS), which has to be considered as a previously healthy biological system (at least in the majority of cases), still regulated by a physiological state, at time of injury. This starting point is particularly helpful for understanding why the number of experimental animal models of TBI is much larger than in neuro-oncology [27]. Neuro-trauma, however, poses several peculiar challenges, both in terms of diagnosis and prognosis: firstly, the range of pathological conditions is very broad and conventional clinical/radiological screening might result in a false negative; secondly, due to involvement of many neural networks and underlying specific cognitive functions, the long term functional outcome might become significantly impaired. For those reasons, identifying markers with the potential to predict the clinical and surgical outcome of TBI could have a dramatic impact in the management of many patients appearing and behaving very similarly at baseline.

Masking of low abundance proteins by high abundance ones is a profound problem that limits the dynamic range of proteomics in TBI patients. In humans, approximately 24,000 genes are translated into an estimated 2 million protein isoforms that may span up to 12 orders of magnitude in abundance in blood, in which proteins originate from hundreds of different cell types. Paradoxically, less than 100 protein biomarkers are routinely measured in blood today [25]. Conventional readouts such as enzyme-linked immunosorbent assay (ELISA) and qualitative Mass Spectroscopy (MS) represented the first proteomic techniques, in which peptides and proteins are recognized either by immunoreactions with specific antibodies or by searching high-quality spectra from protease-specific peptides; both have several constraints in terms of both time and sensitivity [28]. Consequently, in the past, studies resulted that were statistically underpowered, which focused on qualitative or semiquantitative methods for identifying large numbers of proteins in relatively small numbers of brain tissue specimens.

*Identifying biomarkers in neuro-traumatology, the impact of bioengineering*: In recent years, diagnostics have become one of the building blocks of nanomedicine, introducing new methodologies that are empowering clinicians with information that comes from accurate biosensors, devices that combine a biological component with a physicochemical detector used for the recognition of an analyte. Whereas the traditional readout systems mentioned above required large volumes, ultimately diluting the specimens to generate detectable signals at the picomolar range (pM, 10–12 M/L), with single protein or single molecule assays the focus has shifted to the presence or absence of the protein or molecule of interest and the related signal, which is at very low femtomolar concentrations (fM, 10–15 M/L) [29,30,31,32]. To support the development of those analytical methods for detection of relevant biomarker at a micro- or nanometer scale, scientists pursued innovative techniques that are able to reduce the quantities of biological specimens required for diagnostic assays, while scaling down the minimum amount of DNA, RNA, or proteins that can be directly detected. This process led to the acquisition of more comprehensive knowledge regarding the over-expression or under-expression of certain proteins and their physiological or pathological correlations.

This miniaturization proper of nanotechnology has already allowed for the manufacture of portable, hand-held, implantable, or even injectable devices; as a result of their minute size, these devices need less sample or reagent for analysis or operation, resulting in enhanced cost- and time-effectiveness. In the field of proteomic analysis, harnessing the ability to precisely and reproducibly actuate fluids and manipulate bioparticles, such as DNA, cells, and molecules, led to attempts to replicate laboratory bench-top technology on miniature chip-scale devices. For example, some of those techniques, known as point-of-care diagnostic testing, are conceived to allow physicians to diagnose a patient’s conditions more rapidly than conventional lab-based testing and, most importantly, to do it at the bedside.

Single-molecule and single-cell proteomic analysis once hardly conceivable are now increasing their impact on both basic and clinical research. Bioengineering and nanotechnology contributed to an increase in the sensitivity and multiplexing of those methodologies, thus increasing their reliability, rapidity, and throughput. A total of three articles revolve around those techniques; briefly, they provide nuances on single- and multiplexed digital immunoassays developed for recognition of several different proteins of interest in TBI [17,18,21]. Of note, we should mention that some methodology papers were excluded from this systematic review because they failed to provide outcome measures or predictions; the average sensitivity improvement versus conventional ELISA turned out to be >1200-fold, with coefficients of variation of <10% [33].

However, one of the problems that scientists and clinicians have to face with TBI is the type of biological sample used to search predictive biomarkers. This must be easily and rapidly obtained in the early phases following trauma, and of course in the least invasive way. Besides saliva and plasma, which represent the most available specimens at the time of admission to the Emergency Department, a great deal of attention has been focused on CSF, which is thought to show more rapidly and specifically than any other biological tissue all the meaningful changes or imbalances in neural pathways. Ensuring that the CSF samples are properly collected and screened via reversed-phase liquid chromatography (RPLC) and mass spectrometry (MS) entails a specific preparation that can be carried out with different effective techniques [34].

Some of the articles selected for this systematic review provided great insights into how bioengineering is enhancing those techniques, making them faster and more reliable. For instance, Núñez Galindo et al. validated a scalable, automated proteomic pipeline for the sample preparation and proteomic analysis of CSF, enabling increased throughput and robustness for biomarker discovery [18]. In their study, human CSF samples were depleted from abundant proteins and subjected to automated reduction, alkylation, protein digestion, multiplexing labeling, pooling, and sample cleanup in a 96-well-plate format before reversed-phase liquid chromatography tandem MS (RP-LC MS/MS). Specifically, they demonstrated the impact on the CSF proteome coverage of applying the depletion of abundant proteins, which is usually performed on blood plasma or serum samples; by using this methodology to analyze 96 identical CSF samples, they screened, with quantitative accuracy, the individual protein variability of up to 387 proteins [18].

Another example of innovation in quantitative MS is the rapid microwave and magnetic (M^2^) sample preparation: this quantitative methodology for proteomics enables relative protein expression to be correlated with disease progression in neuroinflammatory and TBI models [25,35]. Evans et al. employed this technique in an animal model of mild TBI, confirming that microwave-assisted reduction, plus alkylation, plus digestion of proteins from brain tissue lysates, coupled with magnetic and microwave-assisted isobaric chemical labeling allowed us to identify statistically significant changes in the expression of pivotal biomarkers of neuronal damage. With this method they optimized the identification of myelin basic protein (MBP) and myelin-associated glycoprotein (MAG), which both belong to a family that constitutes the third most abundant proteins in CNS myelin: those proteins areknown products of acute and chronic oligodendrocyte demyelination and are strongly predictive of functional outcome in patients with mild TBI [25].

Also, MicroRNAs have emerged as novel diagnostic biomarkers for various diseases including TBI [26]. Plasma-derived miRNA biomarkers, used in combination with established clinical practices such as imaging, neurocognitive, and motor examinations, have the potential to provide useful biosignatures of TBI, improving the classification of injuries and their management [15]. The serum and CSF levels of miRNA have been investigated as biomarkers for TBI severity and outcome; this approach suggested that some miRNAs play important roles in the pathogenesis and progression of TBI. This research has been implemented in 7 articles identified in this systematic review: 4 clinical and 3 based on experimental animal models. The most important aspect of those studies was the possibility of identifying more severe forms of TBI starting from identical clinical baseline and predicting their early and late outcomes [12,13,14,15,19,20,26].

Finally, one additional methodology deserves mentioning: the study of microvescicles and exosomes in TBI: the attention toward the release of membranous structures such as exosomes, microvesicles, or extracellular vesicles, depending on specific characteristics, including size, composition, and biogenesis by cells into their extracellular environment is justified for several reasons [11]. Besides the miRNA and proteins associated with the cellular responses to TBI events, exosomes can exhibit also an array of biological properties, interacting with other proteins, lipids, and nucleic acids. As such, exosomes and microvescicles can be studied as both diagnostic and causative factors in the progression of TBI [36]. Maneket al. exploited this strategy in patients with severe TBI, and managed to identify in their CSF well known biomarkers, such as αII-spectrin breakdown products, UCH-L1, and GFAP, which are predictive of the clinical progression and development of functional deficits [11]. Those biomarkers reflect the loss of integrity of the cellular structure, including dendrites and axons, the altered brain protein metabolism, and synaptogenesis, respectively; the possibility of screening for circulating exosomes correlated to these biomarkers in several different biological specimens represents a major breakthrough. It can, in fact, be anticipated that, because of their putative role in mediating the secondary brain injury, their presence may serve as a surrogate for biopsies, enabling real-time diagnosis and monitoring of neurodegenerative progression [37].

This systematic review confirmed that the research efforts for identifying predictive biomarkers in TBI are currently evolving into multiple directions, thus increasing the understanding of early stages of TBI and the relationship between specific patterns of biomarkers and the short, medium, and long term outcome of those traumas. Overall, the approaches described in our review allowed researchers to experimentally theorize and, in many cases, to clinically confirm several putative glial and inflammatory biomarkers, which appear to be early (within 24 h from injury) or chronically (over the first 3 months from injury) upregulated. Most of them can be derived from blood, CSF, and even brain tissue at time of surgical intervention, or during invasive intracranial monitoring through external ventricular drains or parechymal microdialysis [38]. Future directions in TBI include exploration of simultaneous multi-marker detection with the use of the abovementioned techniques and experimentation with novel devices to process tiniest amounts of biological specimens collected at time of admission to the Emergency Department [39,40].

So far, an heterogeneous group of polypeptides emerged as promising biomarkers for TBIs of different severities; this group includes structural proteins and enzymes reflecting either glial (S-100β, Tau proteins, GFAP, but also Rho-associated protein kinase 2 and carbonic anhydrase-I) or neuronal (peroxiredoxin-2, synaptosomal-associated protein 25, and microtubule-associated protein 1B) wellbeing or damage, depending on their variation from physiological range [11,12,13,14,15,16,17,18,19,20,21,22,23,24,25,26,41,42,43,44,45]. Since many neurological and behavioral abnormalities observed in patients with TBI may in fact be transient or permanent, and are the visible consequences of the cellular, sub-cellular, and molecular pathological processes and their evolution with time, investigations of animal models still result emphasize the paramount importance of approaching all of them in a more comprehensive way. Due to the complexity and heterogeneity of TBI, as demonstrated in this review, those studies will likely serve as a new ground to better predict the short and long term outcome of patients with very different conditions such as moderate concussion versus devastating brain injuries.

*Limitation of this systematic review*: This article was aimed at describing the state of the art in the application of nanotechnology and biomedical engineering to the field of clinical and functional profiling in neuro-traumatology; however, this introduced two major limitations: the first related to the content of the articles falling within the scope of our research, and the second related to the literature search strategy itself. On one hand, the heterogeneity of the studies included in this review did not allow us to perform any form of metanalysis; as such, it is impossible to tell which biomarker works better, or which sampling and analytical method would be the most appropriate, for any given subtype of TBI. Those questions are yet to be answered by the neuroscience community; although they were not among the aims of this article, should advances in nanotechnology and biomedical engineering be able to contribute to such comparison in the future, they will certainly highlight the importance of the type of research discussed in our paper. On the other hand, the design of this systematic review did not include additional searches of the grey literature (including abstract or conference proceedings) to avoid the bias of including studies not validated by an external thorough peer review process. Also, additional searches regarding planned or ongoing randomized controlled trials or patents protection were not conducted; as such, the following databases have not been interrogated: ClinicalTrial.gov Database, United States Patent and Trademark Office Database, and European Patent Office Databases, whereas we estimate a crossover for many articles that would have been identified through those additional searches, and therefore we believe that we did not lose any relevant scientific information; this choice implied our inability to collect other information that could show where the research field is currently heading. For instance, as a result of this decision we failed to identify which countries are investing the most or are lined up to obtain the highest return on their investments in nanotechnology and biomedical engineering. Nonetheless, this limitation was identified and accepted early on in the initial stages of our study, and eventually deemed not relevant for the research question stated at the beginning of this article, which was meant to solely define a possible role for those innovative technologies in providing clinically meaningful biomarkers with a potential to improve the management of TBI patients.

## 5. Conclusions

In conclusion, despite the still limited available results gathered from the innovative methods for proteomic profiling described in this review, the directions of future development pertaining to this field seem promising. Considering the outstanding technical challenges of identifying reliable biosignatures for TBI and the mixed nature of studies herein described (single cells proteomics, biofilms, sensors, etc.), our opinion is that the clinical application of those discoveries will allow us to gain confidence in the use of those advanced neuromonitoring modalities with a potential dramatic improvement in the management of TBI patients.

## Figures and Tables

**Table 1:** Nanotech and Biomedical Engineering advances in proteomic profiling: clinical studies of TBI patients.

Methodologies and References	Findings
*Microvescicles/Exosome*	
Manek, R., et al., 2017 [11]	Using targeted immunoblotting approach, several known TBI biomarkers such as αII-spectrin breakdown products, GFAP, and UCH-L1 were found in higher concentrations in microvescicles/exosomes from TBI CSF than their counterparts from control CSF.
*MicroRNA*	
Di Pietro, V., et al., 2017 [12]	Using a real time PCR/MicroRNA assay, early downregulation of miR-425-5p and miR-502 in moderate TBI, and upregulation of miR-21 and miR-335 in patients with severe TBI, were demonstrated. In addition, miR-425-5p and miR-21 were demonstrated to be strong predictors of the 6-month outcome at ultra-early (T0-1 h) and early time points (T4-12 h).
Bhomia, M., et al., 2016 [13]	Using a real time PCR/Micro RNA assay, accurate biomarkers of TBI were identified: miR-195, miR-451, miR-92a, miR-486, miR-505, miR-362, and miR-20a. The computational analysis of the 30 genes identified as direct targets for the miRNA candidates listed above revealed involvement of important neurological pathways (i.e., G protein-coupled receptor signaling, GABA receptor signaling, neuropathic pain signaling, etc.).
Yang, T., et al., 2016 [14]	Using a real time PCR/Micro RNA assay miR-93, miR-191, and miR-499 emerged as plasma biomarkers to distinguish mild TBI patients from healthy controls.
Redell, J.B., et al., 2010 [15]	Using a real time PCR/Micro RNA assay miR-16, miR-92a, and miR-765 were identified as good markers of severe TBI.
*MALDI Mass Spectrometry*	
Connor, D.E., Jr.; et al., 2017 [16]	Using a MALDI MS approach, a consistent CSF elevation of carbonic anhydrase-I (CA-I) and peroxiredoxin-2 (Prx-2), both α and β chains of hemoglobin, with concurrent depletion of serotransferrin (Tf) and N-terminal haptoglobin (Hp), emerged as a useful combination of biomarkers for the prediction of severity and prognosis following TBI.
*Multiplexing and Immunoassays*	
Rubenstein, R., et al., 2017 [17]	Using an ultra-high sensitivity, laser-based, immunoassay, multi-arrayed fiberoptics conjugated with rolling circle amplification, this study demonstrated that plasma P-tau levels and the P-tau/T-tau ratio outperformed T-tau level as diagnostic and prognostic biomarkers for acute TBI. On the other hand, compared with T-tau levels alone, P-tau levels and P-tau/T-tau ratios show more robust and sustained elevations among patients with chronic TBI.
Núñez Galindo, A., et al., 2015 [18]	Using a scalable automated proteomic pipeline known as ASAP(2) for the sample preparation and proteomic analysis of CSF and plasma in TBI patients, this study showed increased throughput and robustness for biomarker discovery, enabling proteome coverage consistency (up to 387 proteins screened), quantitative accuracy, and detection of individual protein variability.

**Table 2:** Nanotech and Biomedical Engineering advances in proteomic profiling: animal models of TBI.

Reference	Model of TBI	Findings
Sajja, V.S.S.S., et al., 2017 [19]	Murine model of mild to moderate blunt TBI	Plasma levels of miR-127, as well as lipid profiling with decreased C18 fatty acid chains of sphingomyelins and increased ceramide levels in TBI models compared to controls.
Chandran, R., et al., 2017 [20]	Mice models of mild TBI	Axon guidance, calcium signaling, and various synaptic pathways such as dopaminergic, GABAergic, glutamatergic, and cholinergic synapse pathways appear significantly affected by the miRNAs modulated at both 24 h and 7 days post-injury (miR-27a, miR-150, miR-155, miR-222, miR-223 and miR-449a, miR-744, and miR-874).
Wofford, K.L., et al., 2017 [21]	Swine model of mild to severe TBI	Single cell quantitative analysis showed that neuronal trauma rapidly activates microglia in a highly localized manner, being restrained to regions proximal to individual injured neurons (trauma-induced plasma membrane disruption) erve as epicenters of acute reactivity.
Kobeissy, F.H., et al., 2017 [22]	Rat models of moderate to severe TBI	Gene ontology analysis of the proteomic data allowed us to categorize the proteins by molecular function, biological process, and cellular localization, showing alterations in several proteins related to inflammatory responses and oxidative stress in both acute (1 day) and subacute (7 days) periods post-TBI. Moreover, a differential upregulation of neuroprotective proteins involved in cellular functions such as neurite growth, regeneration, and axonal guidance was shown at 7 days post-TBI.
Zhang, P., et al., 2016 [23]	Rat models of diffuse axonal injury	Among biomarkers for diffuse axonal injury, identified by iTRAQ coupled liquid chromatography/mass spectroscopy, four proteins (citrate synthase, synaptosomal-associated protein 25 (Snap25), microtubule-associated protein 1B (MAP1B), and Rho-associated protein kinase 2 (Rock2)) were successfully validated by subsequent Western blot and immunohistochemistry analyses.
Haselwood, B.A., et al., 2015 [24]	Rabbit models of mild to moderate TBI	Using electrochemical impedance techniques for point-of-care TBI diagnosis, it was possible to detect sustained blood elevation of norepinephrine concentrations, known to negatively relate to long-term outcomes in TBI, with lower limit of detection in the range of pg/mL.
Evans, T.M., et al., 2014 [25]	Mouse models of mild TBI	M^2^ proteomic analysis revealed statistically significant changes in the expression of myelin basic protein (MBP) and myelin-associated glycoprotein (MAG), both well know biomarkers of neuronal damage, at 1, 7, and 30 days post-TBI. MAG, αII-spectrin (SPNA2) and neurofilament light (NEFL) expression at 30 days post-TBI resulted related to functional outcome.
Balakathiresan, N., et al., 2012 [26]	Rat models of moderate blunt TBI	Elevated plasma and CSF levels of miRNA let-7i appear immediately after blast wave exposure. Of note, miR-let-7i seems associated with the expression of proteins and inflammatory cytokines, including S100β and UCH-L1, already investigated as biomarkers for TBI.
